# Future-proofing ecosystem restoration through enhancing adaptive capacity

**DOI:** 10.1038/s42003-023-04736-y

**Published:** 2023-04-07

**Authors:** Marina Frietsch, Jacqueline Loos, Katharina Löhr, Stefan Sieber, Joern Fischer

**Affiliations:** 1 Leuphana University, Social-Ecological Systems Institute, Faculty of Sustainability, Universitätsallee 1, 21335 Lüneburg, Germany; 2 University of Rwanda, Center of Excellence in Biodiversity and Natural Resource Management, KN 7 Ave, Kigali, Rwanda; 3 Leuphana University, Institute of Ecology, Faculty of Sustainability, Universitätsallee 1, 21335 Lüneburg, Germany; 4 Leibniz Centre for Agricultural Landscape Research (ZALF), Eberswalder Straße 85, 15374 Müncheberg, Germany; 5 Humboldt Universität zu Berlin, Thaer-Institute of Agricultural and Horticultural Sciences, Urban Plant Ecophysiology, Lentzeallee 55/57, 14195 Berlin, Germany; 6 Humboldt Universität zu Berlin, Thaer-Institute of Agricultural and Horticultural Sciences, Resource Economics, Unter den Linden 6, 10099 Berlin, Germany

**Keywords:** Restoration ecology, Ecosystem services

## Abstract

Social-ecological ecosystem restoration involves interacting challenges, including climate change, resource overexploitation and political instability. To prepare for these and other emerging threats, we synthesized key restoration and social-ecological systems literature and derived three guiding themes that can help to enhance the adaptive capacity of restoration sites: (i) work with the existing system, (ii) create self-sustaining, adaptive systems, and (iii) foster diversity and participation. We propose a two-step approach and provide an example from Rwanda detailing the application of these principles. While site-specific activities have to be designed and implemented by local practitioners, our synthesis can guide forward-thinking restoration practice.

## Abstract (web summary)

This Perspective piece outlines potential avenues to support ecosystem restoration projects into the future.

## Introduction

The restoration of degraded ecosystems is increasingly recognized as a key strategy to respond to climate change, biodiversity decline, and associated ecological and social challenges^1,2^. Worldwide, many initiatives from local to global scales contribute to ecosystem restoration^3,4^, and the United Nations declared 2021–2030 the “Decade on Ecosystem Restoration”^5^. Ecosystem restoration can be defined as the “process of halting and reversing the degradation of ecosystems”^6^. In practice, restoration encompasses diverse activities that range from revegetation^7^ through interventions to restore species composition, ecosystem structure or function^8^, to approaches that aim for multifunctional landscapes such as forest landscape restoration^9^. Increasingly, restoration is no longer seen as a purely ecological task but rather as a social-ecological endeavour^10,11^ that seeks to restore inherent ecosystem values as well as provide goods and services to humanity^12,13^. Defined this way, ecosystem restoration needs to consider species composition, ecosystem functions and services, as well as human well-being.

Restoration activities inherently need to grapple not only with connections across space, but also with connections through time: restoration is informed by the past but created for the future, while drawing on the knowledge of today. Because the world is rapidly changing, past reference states might significantly differ from biophysical and also social conditions that shape a specific site now, let alone in 50 or 100 years^14,15^. In some cases, novel or hybrid ecosystems might emerge that are characterized by significantly altered abiotic conditions and new, unprecedented species assemblages^16^. Rapid and partly unpredictable social-ecological change thus makes ecosystem restoration particularly complex, and also influences the context in which restoration activities take place^17^.

Despite their importance, three well-known challenges are commonly neglected in the design of restoration projects: (1) climate change, (2) overexploitation of resources, and (3) political instability. While some aspects of all three challenges have been discussed in recent restoration literature^18–21^, restoration practice still lags behind in consistently taking measures to safeguard restoration sites against future threats^15,20,22^. As a result, how climate change, overexploitation of resources, and political instability affect restoration sites in the short and medium term remains underexplored. In addition, interactions among these challenges—and perhaps other contemporary or emerging social-ecological changes—might result in new types of compounding threats for the viability of current and planned restoration efforts.

In this perspective article, we discuss how these three challenges and their interactions could disrupt, impede, or undermine ecosystem restoration. Based on this, we provide tangible suggestions for ways forward. Specifically, we (1) generate a concise synthesis of key principles from restoration and social-ecological systems literature, (2) introduce a two-step approach detailing how the resulting three guiding themes can be applied to restoration sites, and (3) illustrate via a case study on western Rwanda how the application of this approach can help restoration sites prepare for emerging challenges that might increasingly influence restoration in the future. In combination, our discussion of potential future threats, integration of different bodies of literature and operationalization of the resulting guiding principles provide a novel approach that can guide forward-thinking restoration practice.

## Three key challenges for long-term successful restoration

**Fig. 1:**
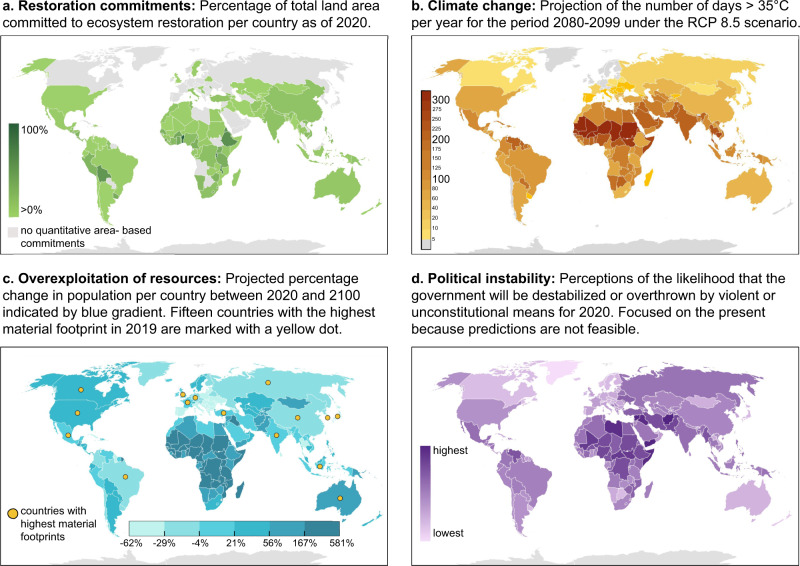
Global patterns of restoration commitments, projected effects of climate change, overexploitation of resources, and political instability. The areas where many restoration activities are being implemented or planned (**a**) are also disproportionately affected by climate change (**b**), overexploitation of resources (**c**), and political instability (**d**). Data sources: **a** Restoration commitments from the Global Restoration Commitments Database by the PBL Netherlands Environmental Assessment Agency^90^. **b** Climate change represented by the number of days above 35 °C in the period 2080–2099 from the Climate Impact Lab.^85, 91^. **c** Human population growth from the United Nations World Population Prospects 2019^49^ and national material footprint from the United Nations Environment Programme International Resource Panel Global Material Flows Database^92^ (a list of the 15 countries is provided in Supplementary Table 1); **d** Political instability from the Worldwide Governance Indicators project by the World Bank^93^.

Globally, locations predicted to experience severe climate change, overexploitation of resources, and political instability broadly coincide with locations earmarked for ambitious restoration activities (Fig. 1). Drawing on examples from around the world, we illustrate some of the many impacts that these challenges can have on social-ecological systems in general, and that they may have on restoration sites in particular.

### Climate change

Climate change challenges ecosystem restoration by shaping future biophysical conditions in ways that are difficult to predict and, in some cases, may result in entirely unknown system constellations^16^. Climate models project that temperatures will rise, precipitation patterns will change, sea levels will rise, and the occurrence of extreme weather events will increase over the coming decades^23,24^. Notably, climate change is projected to have above-average impacts on regions with many restored and pledged restoration sites—as indicated for example by the number of extremely hot days predicted for the future (Fig. 1b).

Climate, directly and indirectly, influences ecosystem structure and processes, as well as the distribution of species and ecosystems^25^. Changes in climate might cause biome and habitat shifts^26,27^. At a coarse resolution, biome boundaries might shift^28^ either gradually or abruptly, depending on the scale and type of climate change-induced pressure^29^. For example, the boundaries of the Sahel, where the Great Green Wall (one of the world’s most ambitious restoration projects) is currently being implemented^3^, are anticipated to shift southwards as a response to increasing temperatures, changes in precipitation patterns, and general drying in the region^28,29^. Such biome shifts could have major effects on restoration outcomes because ecological conditions will change over vast areas of land. At a finer resolution, climatic changes will cause the ranges of individual species to shift, generally to higher latitudes and higher altitudes^30^. Such changes in habitat suitability have major implications for species selection in restoration, and require the consideration of different timescales in restoration practice. Besides shifts in the location of biomes and habitats, the spatial extent of these will also change in response to climate change, and completely new systems with unknown ecological outcomes might emerge in the process^31^. Unless restoration is planned with climate change in mind, species newly planted for ecosystem restoration could fail to keep pace with associated shifts in geographical range^32^.

Another aspect of how climate change might influence restoration sites is the effect of more frequent and intense extreme weather events^15,23^. Extreme temperatures, precipitation, floods, droughts, winds, or wildfires have the potential to destroy vast areas of biomass at once. For example, the 2021 wildfires in California, United States of America, burned almost 1 million ha^33^. Yet, California is considered a priority area for ecosystem restoration with a high potential to safeguard biodiversity and mitigate climate change^34^. Extreme weather events can irreversibly alter the structure of ecosystems and cause the decline or even local extirpation of species^35^. Notably, changes in patterns of extreme weather events are acknowledged to impact ecosystem functioning more strongly than shifts in average conditions^36^. Combinations of gradual changes in temperature and precipitation as well as more frequent extreme weather events thus could significantly undermine restoration efforts locally because planted species may not grow as expected, or because extreme weather events could destroy restoration areas.

### Overexploitation of resources

Overconsumption as well as human population growth challenge ecosystem restoration by increasing global and local demand for natural resources, potentially triggering their overexploitation. Intensifying pressure on ecosystems therefore is both the reason why ecosystem restoration is so urgently needed, as well as a potential threat to restored ecosystems^37,38^. In addition, overexploitation aggravates competition for space between long-term restoration projects and resource extraction that benefits people in the short term^39,40^. In most high-income countries, the human population is projected to only grow moderately or may even decline in the coming decades (Fig. 1c). However, excessive per capita consumption in these countries results in a disproportionate demand for resources^41^. Consumption-related environmental impacts caused by high-income countries are estimated to be three to six times larger than those of low-income countries^42^. Often, these impacts are outsourced to middle- and low-income regions^43,44^. South American countries, for example, are the largest producers of soybeans which are used as livestock feed in the European Union. Large-scale soy production for the international market in countries such as Brazil and Argentina has resulted in the loss of primary forests, the expansion of monocultures^45^, and grassland conversion, while providing no economic benefits to society at large^46^. Such telecouplings (i.e., interactions among social and ecological phenomena across large distances^11,47^ and ecologically unequal exchange (i.e., asymmetric exchange of biophysical resources from poorer to richer countries^43^ increasingly shift environmental burdens to poorer countries, and thus to many of the nations that have committed themselves to ecosystem restoration (Fig. 1a).

In addition to excessive consumption in the Global North, human population growth (especially in the Global South) can also fuel the overexploitation and degradation of ecosystems^38^ and can be an important driver of deforestation^48^. The world population is projected to grow from nearly 8 billion today to 10.9 billion in 2100^49^. As with climate change, rapid population growth is projected to occur in many countries where ambitious restoration projects are being implemented or planned, such as Pakistan, Bolivia, and Nigeria (Fig. 1c). In Madagascar, the co-occurrence of human population growth and forest loss is clearly evident: a rising demand for food and energy has caused the ongoing clearance of formerly forested land for agricultural production and biomass extraction. Between 1950 and 2000, Madagascar’s population grew from 4.1 million to 15.7 million^49^. In the same time period, 40% of forest cover was lost, leading to losses of biodiversity and ecosystem services, as well as causing soil degradation and increased carbon dioxide emissions^50,51^. Similarly, population growth played a central role in deforestation in Malawi^52^, where a 1% increase in the human population was related to a deforestation rate of 2.7%^38^. Today, Madagascar and Malawi are among the countries with the highest projected population growth in the coming decades^49^; but they have also pledged to restore close to 7% and 50%, respectively, of their land by 2030^4^.

Notably, increasing resource demands caused by high per capita consumption and human population growth are met not only by land-use change, but also by intensifying production on existing agricultural land^38^. In the Brazilian Amazon, for example, intensification was induced by local population growth and associated growing demand for food^53^. However, in many cases, agricultural intensification fails to achieve its goal of delivering higher levels of ecosystem services or human well-being, especially when it is driven by human population growth^54^. In the Brazilian example, agricultural intensification in fact caused a reduction in cassava yield while simultaneously increasing labour and decreasing household incomes^53^.

In combination, high per capita resource demand in high-income countries combined with growing demand for essential ecosystem services by a growing population in low-income countries could jeopardize restoration activities: growing demand, necessity, and resource scarcity might drive people to prioritize the short-term gains of extracting resources—and potentially degrading restoration sites in the process—over the long-term benefits of restoration. Moreover, mosaic restoration that integrates trees into mixed-use landscapes, especially agricultural lands, is a key restoration strategy that seeks to generate diverse benefits for both ecosystems and humans^55^. The ongoing simplification of agricultural landscapes driven by a desire to increase agricultural yields, however, runs counter to mosaic restoration^56^.

### Political instability

Political instability challenges ecosystem restoration by destabilizing structures that are vital for the implementation and ongoing management of restoration projects (Rai et al.^57^). We define political instability as the absence of orderly transfers of government power and failure to maintain the rule of law, leading to social unrest, tensions, and conflicts at an international, national, or regional level. Because political instability is inherently uncertain, future developments in political stability are difficult to predict^58,59^. However, many of the priority areas for restoration have experienced political instability in the past^58^ or are unstable today (Fig. 1d). It thus seems reasonable to expect that these areas also could be vulnerable to ongoing political instability in the coming years and decades^60,61^.

To reach its full potential, ecosystem restoration hinges on a minimum level of stable political and societal conditions. This includes the protection of restoration sites through legal tenure arrangements^62,63^, as well as long-term political and economic commitments by major public and private stakeholders^13,64^. In areas where such conditions are missing, the degradation of restoration sites is probable. When political instability undermines the rule of law, high levels of corruption and impunity can result in illegal destruction of ecosystems and even threaten the lives of environmental activists. For example, many murders of environmental defenders became public in recent years, especially in countries across Latin America^65^. Case studies in Nepal, Sri Lanka, Peru, and Côte d’Ivoire further show that deforestation rates had increased in the aftermath of armed conflicts when resource demands were high, and when political instability resulted in weak enforcement of environmental regulations^66^.

In the worst case, political instability can result in armed conflict. This can trigger human displacement, forced reliance on natural resources, uncontrolled resource exploitation, and subsequently biodiversity loss^67^. For example, an analysis of the effect of armed conflicts in forests in Colombia between 1992 and 2015 revealed that conflict areas were eight times more likely to undergo deforestation relative to average deforestation rates. The main drivers included efforts to finance the conflict through illegal agricultural production, mining, and logging, as well as insecure land tenure and unstable political institutions that paved the way for land grabbing^68^. Similarly, in Myanmar, the world’s longest civil war and recent political transitions have caused the degradation of ecosystems due to weak land tenure, economic pressures, internal displacement, and other associated factors^69^. Based on these examples, armed conflicts can be expected to degrade or even destroy restoration sites, and the effects are difficult to anticipate and control. For example, the civil war in Ethiopia’s Tigray region which erupted in 2020 substantially overlaps with many of Ethiopia’s key restoration sites in the Great Green Wall^3^ – but at present, consequences of the conflict for these sites remain unknown.

### Additional challenges, interactions, and unforeseen surprises

While climate change, overexploitation of resources, and political instability are central challenges ecosystem restoration will need to face, they are far from being the only social-ecological pressures. Other potential ecological risks are related to (1) the spread of invasive species that can significantly change the composition of ecological communities, result in biotic homogenization^70^, or cause the local extinction of species^71^; (2) habitat fragmentation that only allows for small, isolated restoration sites where biodiversity levels are low and key ecosystem functions are impaired (Haddad et al.^72^); and (3) the pollution of air, water, and soil that can harm organisms and reduce biodiversity^71^. Other social risks include poverty, structural inequities, and a lack of environmental justice^19^, which can limit community commitment to ecosystem restoration and thereby indirectly cause the overexploitation of restoration sites. Finally, conflicting expectations regarding restoration sites can slow down or inhibit the implementation of restoration activities^22,55^ or reinforce social inequalities^19,64^ if stakeholders cannot agree on common goals. Thus, while climate change, overexploitation of resources, and political instability are some of the largest and most visible challenges to the future of restoration sites, they underpin and interact with many other additional challenges. Interactions among possible future challenges to restoration sites are inherently difficult to anticipate and might lead to numerous unforeseen surprises such as the crossing of social-ecological system tipping points. An example of how climate change, overexploitation of resources, and political instability are interconnected in Rwanda, and how this affects ecosystem restoration activities, is provided in Box 1.

Box 1 Ecosystem degradation and restoration in the case of Rwanda The case of Rwanda illustrates the interactions between political instability, redistribution of human pressures due to migration, causing the overexploitation of resources, and climate change in a restoration context across time. Over the past decades, a complex interplay of societal and political factors has resulted in the large-scale degradation of ecosystems in all parts of the country. Unstable political conditions^94^, short-sighted protected area governance^95^, armed conflicts in the 1960s, and a civil war culminating in the genocide against the Tutsi in 1994 caused habitat fragmentation, unregulated resource extraction, biodiversity loss, the killing of endangered animals, and ecosystem pollution^96^. Protected areas experienced disproportionate forest loss following the settlement of refugees^96, 97^ and paramilitary militias^94, 95^ within or close to their borders, with some protected areas losing up to two-thirds of their historical extent^96^. The breakdown in law and order that accompanied the armed conflicts interrupted research and conservation activities, resulted in higher poaching rates, loss of lives of conservationists^94, 96^, and declining conservation funding^94^.

In the aftermath of the civil war, fast-growing non-native plantation forests were established throughout Rwanda^96, 98, 99^. These early restoration efforts focused on establishing woodlots or adopting agroforestry practices due to land scarcity^98^. While these activities provide short-term benefits for local communities such as fuelwood, they have resulted in patchy forests with relatively low biodiversity^98^ and degrading soils^99^. It also remains unclear how the many non-native monocultures will perform under a changing climate.

Despite these limitations of early restoration efforts in particular, today Rwanda is considered one of the world’s ecosystem restoration leaders^9^, and has pledged to restore more than 80% of its terrestrial area. However, in order to respond to ongoing climate change, human population growth, increasing resource demands, and the risk of renewed political instability in the coming decades, Rwanda will need to increase the adaptive capacity of its restoration sites. In the case of Rwanda, this might involve planting a mix of species that respond well to changing climatic conditions, further refining agroforestry approaches that benefit both biodiversity and a growing human population, as well as decentralizing restoration responsibility to safeguard restoration sites even in the event of political turmoil.

## The way forward

When planning ecosystem restoration, it is important to consider the challenges laid out above. Already, there are numerous suggestions for how to design sustainable, adaptive systems that can be applied to restoration. Globally applicable restoration guidelines date back to 2012, when Keenleyside et al. argued for restoration of Protected Areas to be effective, efficient, and engaging. Partly overlapping principles have since been put forward by Suding et al.^73^; while the most recent, comprehensive lists of principles were published by Gann et al.^13^, Gichuki et al.^74^, and FAO et al.^75^. Similarly, numerous principles have been proposed to maintain the adaptive capacity of production landscapes^76^ or social-ecological systems in general^77–79^.

To distil a tangible set of usable suggestions from the burgeoning lists of existing principles, we focus on two key bodies of literature that provide guidance on how to design and manage restoration sites and navigate complex human-environment relations, respectively. Specifically, (i) restoration literature and (ii) social-ecological systems literature both put forward diverse sets of principles that are useful, but that have not been integrated to date. Restoration principles often focus on ecological and design aspects of restoration, and often emphasize the connection between local actions and the larger landscape^13,73–75^. By contrast, in social-ecological systems science, there is a rich literature on resilience—i.e., the degree to which a system can cope with changing conditions while retaining key elements of structure, function, and identity^80,81^—which highlights social capital and the need for a deep understanding of system complexity^77–79^.

Despite their possible utility, the proliferation of theory-driven principles can be difficult to operationalize for restoration practitioners, and as such does not automatically translate into practical implementation on the ground. As a consequence, adequate responses to future uncertainties (such as the three threats outlined above) are still not sufficiently well anchored in restoration practice^15,20,22^. We argue that while theoretical considerations on restoration, system complexity and resilience abound, there is a lack of practical skills, mechanisms, strategies, and institutional structures that support system components in restoration sites (e.g., actors, species, ecological communities) in adjusting to changing environmental and socio-economic conditions. As such, there is a need to further enhance the adaptive capacity of restoration projects^81–84^.

To help navigate the myriad of existing principles and provide tangible guidance on how the adaptive capacity of restoration sites can be increased by integrating insights from restoration and resilience literature, we offer a two-fold contribution. First, we draw on 52 recognized restoration and resilience principles, and distil these into three core guiding themes for how to respond to the specific threats outlined above. Second, we demonstrate how these three themes can be operationalized in practice, and illustrate our approach by applying it to restoration in western Rwanda.

For the synthesis of restoration and resilience principles, we selected seven influential publications by scientists and organisations that together encompass a very broad range of principles that are relevant for social-ecological restoration contexts (for restoration principles, see ref. ^13,73–75^; for resilience principles, see ref. ^77–79^.). For restoration, we focused on the most recent, comprehensive lists of principles. We then iteratively coded these principles into three themes (Table 1). In highly simplified (but usable) terms, the 52 principles can be boiled down to three main themes: (1) work with the existing system, (2) create self-sustaining, adaptive systems, and (3) foster diversity and participation.

**Table 1:** Principles put forward by different sources to support successful ecosystem restoration and enhance resilience.

Restoration Principles	Resilience Principles
Suding et al. 2015^73^	Walker & Salt 2006^77^
Increase ecological integrity *□*	Promote and sustain diversity ☆
Establish long-term sustainable systems *□* ○	Embrace and work with ecological variability *□* ☆
Learn from the past and plan for the future *□*	Maintain and create modularity ○
Benefit and engage society *□* ☆	Acknowledge slow variables *□*
Gann et al. 2019^13^	Tighten the strength of feedbacks *□* ○
Engage stakeholders *□* ☆	Strengthen social capital *□* ☆
Draw on many types of knowledge ☆	Emphasize innovation ☆
Relate to native reference ecosystems while considering environmental change *□*	Create redundancy in governance ☆
Support ecosystem recovery processes *□* ○	Include unpriced ecosystem services *□* ☆
Assess against clear goals using measurable indicators ○	Biggs et al. 2012^78^
Seek the highest level of recovery possible *□*	Maintain diversity and redundancy ☆
Gain cumulative value when applied at large scales *□* ○	Manage connectivity ○
Design activities as part of a restorative continuum *□* ☆	Manage slow variables and feedback *□*
Gichuki et al. 2019^74^	Foster an understanding of social-ecological systems as complex adaptive systems ○
Focus on landscapes *□*	Encourage learning and experimentation ☆
Maintain and enhance natural ecosystems *□*	Broaden participation ☆
Engage stakeholders, support participatory governance ☆	Promote polycentric governance systems ☆
Tailor to local conditions *□*	Carpenter et al. 2012^79^
Restore multiple functions for multiple benefits ☆	Promote diversity ☆
Manage adaptively for long-term resilience ○	Create modularity ○
FAO, IUCN & CEM 2021^75^	Manage openness ○
Contribute to the SDGs and the Rio Conventions ☆	Maintain reserves ○ ☆
Promote inclusive and participatory governance ○ ☆	Manage feedbacks *□*
Include a continuum of restorative activities ☆	Enable polycentric governance by nesting systems ☆
Benefit nature and people ○	Conduct monitoring ○
Address causes of degradation *□*	Promote leadership and trust ☆
Integrate different types of knowledge ☆	
Establish well-defined and measurable goals ○	
Tailor to local contexts while considering the larger landscape *□*	
Include management and monitoring ○	
Ensure an enabling policy environment ○	

We iteratively assigned these principles to three core themes: work with the existing system = □; create self-sustaining systems = ○; foster diversity and participation = ☆.

First, *working with the existing system* means considering restoration sites as social-ecological systems and tailoring restoration activities to local contexts, including site-specific ecological variability and drivers of degradation. Second, *creating self-sustaining, adaptive systems* entails promoting polycentric governance systems, managing feedbacks, and monitoring and responding to developments in restoration sites over time. Third, *fostering diversity and participation* in a given social-ecological restoration system implies strengthening social capital, encouraging exchange and innovation, and promoting functional and response diversity as well as maintaining suitable levels of modularity. All three guiding themes need to be applied at all levels of restoration action, and should consider both the ecological and social components of a given social-ecological restoration system. Ideally, the guiding principles will inform the scoping and design of restoration projects as well as their ongoing management.

**Fig. 2:**
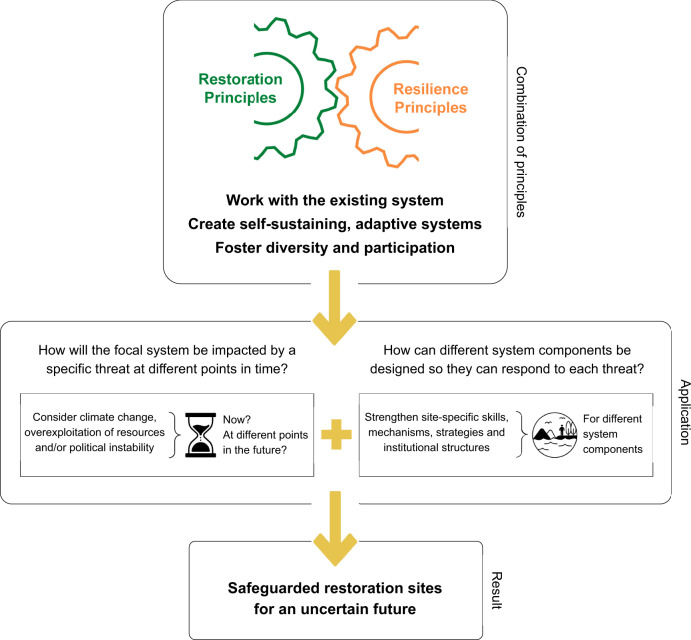
Safeguarding restoration sites by integrating restoration and resilience principles. Three high-level guiding themes for future-proof restoration practice were derived from the synthesis of 52 existing restoration and resilience principles (see Table 1). These guiding principles can be applied to restoration projects to facilitate the development of site-specific skills, mechanisms, strategies, and institutional structures that enhance the restoration system’s adaptive capacity.

To integrate restoration and resilience principles in practice and create restoration sites that are well-equipped to face climate change, overexploitation of resources, political instability, and other unexpected threats, we propose a two-step approach (Fig. 2). The first step is an initial assessment of the impacts each threat would likely have on a specific site at different points in time. Meaningful timeframes will differ across restoration sites; for example, it could mean to consider the present, 30 years and 100 years in the future. As a second step, each of the three guiding themes can be applied to formulate specific activities that can strengthen skills, mechanisms, strategies, and institutional structures that support different system components in adjusting to each threat. Relevant system components might include people living in the restoration landscape, landowners, restoration practitioners, governance bodies and formal institutions, currently occurring ecological communities, and species planted within the scope of restoration activities.

We suggest these two steps because they help to break down the myriad of existing high-level principles into concrete actions to be carried out within a given social-ecological restoration project. The site-specific skills, mechanisms, strategies, and institutional structures that are strengthened in the process will, in turn, enhance the adaptive capacity of the social-ecological restoration site. Restoration projects designed and managed in this way thus can be expected to have an enhanced capacity to respond to climate change, overexploitation of resources, and political instability.

### Practical application

**Fig. 3:**
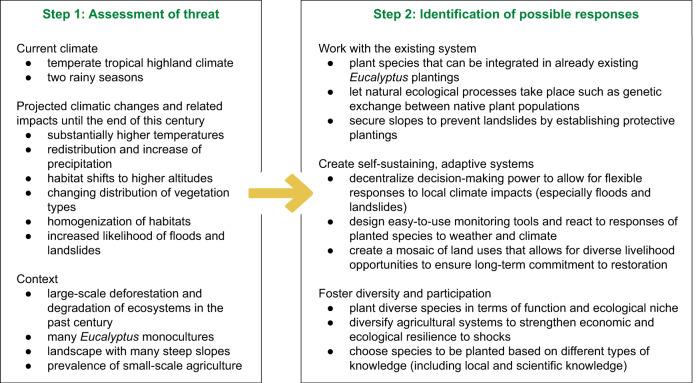
Exemplary application of the three guiding themes to the threat of climate change in western Rwanda. In practice, the three guiding principles can be applied to any possible threat. Here, we apply it to effects of climate change on the Albertine Rift in western Rwanda. In a first step, the current context and the effects of the focal threat need to be understood. In a second step, possible context-specific responses based on the three guiding principles can be formulated.

To illustrate what this approach may look like in practice, we use the example of the Albertine Rift in western Rwanda. We specifically consider climate change in this instance, noting that a similar rationale could also be applied to political instability or resource exploitation. Major climatic changes expected for the Albertine Rift relate to a substantially warmer climate^85^, the redistribution and increase of precipitation, altitudinal habitat shifts^86^, a changing distribution of vegetation types and homogenization of habitats, and an increasing likelihood of floods and landslides^87^. Combining these potential changes with the three themes suggested above results in 12 concrete activities that can be embedded in restoration projects to increase their adaptive capacity (Fig. 3). Notably, these activities are examples only. Ideally, a combined group of scientists, local practitioners and policy makers should design suitable activities in a collaborative process.

While the suggested strategies in Fig. 3 are by no means complete, they illustrate how professionals and communities involved in restoration in western Rwanda could potentially increase the adaptive capacity of restoration sites to climate change by drawing on recognized restoration and resilience principles. Similarly, in the case of overexploitation of resources, viable strategies might include, for example, creating a mosaic of land uses that allow for diverse livelihood opportunities, designing incentives for the long-term commitment to restoration on different organisational levels, safeguarding restoration sites via contextually appropriate land tenure arrangements, and making telecouplings visible that connect restoration sites with actors elsewhere. To safeguard restoration sites against possible incidents of political instability, possible strategies might be decentralizing power and decision-making authority to local and regional levels, being sensitive to existing power imbalances and inequalities in communities where restoration takes place, organizing restoration as independent as possible from temporary political power structures—especially when the political system is prone to quick, fundamental changes. As noted above, these are general suggestions only; collaborative processes would be required to generate an authoritative list of context-specific adaptive responses that also take into account possible interactions among potential threats.

## Conclusion

In conclusion, climate change, resource overexploitation and political instability individually and in combination generate major uncertainty for restoration projects in many parts of the world. Restoration and social-ecological systems literature can guide forward-thinking restoration practice to address these threats by strengthening site-specific skills, mechanisms, strategies, and institutional structures that enhance a system’s adaptive capacity. Drawing on a combination of resilience and restoration principles is valuable not only when applied to the three threats identified in this paper, but can also support the development of measures aiming to enhance the resilience of restoration sites to other site-specific, interconnected threats. Across both ecological and social realms, the particular ways to enhance the capability of a restoration site to adjust to change will vary. Hence, it is central to bring together researchers, practitioners, policy-makers and the people living at restoration sites to exchange knowledge and align restoration practice with local realities^22,88,89^. This way, restoration sites will stand the best chance of benefitting both nature and people into the long-term future.

### Reporting summary

Further information on research design is available in the Nature Portfolio Reporting Summary linked to this article.

## Supplementary information

Supplementary Information

Reporting Summary
