# Supplementary material for: Future-proofing ecosystem restoration through enhancing adaptive capacity
Source: Commun Biol. 2023 Apr 7;6:377. doi: 10.1038/s42003-023-04736-y (PMC10082013; doi:10.1038/s42003-023-04736-y)
Supplement: Supplementary file 1 — Supplementary Information [file 42003_2023_4736_MOESM1_ESM.pdf]

Supplementary Table 1

The 15 countries with the highest material footprint in 2019 according to the United Nations Environment Programme International Resource Panel Global Material Flows Database [90]. The material footprint attributes global material extraction to domestic final demand of a country.

**Rank**	**Country**
1	China
2	United States of America
3	India
4	Brazil
5	Japan
6	Indonesia
7	Germany
8	Turkey
9	Mexico
10	Russia
11	Canada
12	United Kingdom
13	Australia
14	South Korea
15	France
